# Solanine Induces Mitochondria-Mediated Apoptosis in Human Pancreatic Cancer Cells

**DOI:** 10.1155/2014/805926

**Published:** 2014-05-11

**Authors:** Hongwei Sun, Chongqing Lv, Longlong Yang, Yingxiu Wang, Qingshun Zhang, Suhui Yu, Hongru Kong, Meng Wang, Jianming Xie, Chunwu Zhang, Mengtao Zhou

**Affiliations:** ^1^Department of Surgery, First Affiliated Hospital of Wenzhou Medical University, Wenzhou 325000, China; ^2^Wenzhou Key Laboratory of Surgery, Department of Surgery, First Affiliated Hospital of Wenzhou Medical University, Wenzhou 325000, China

## Abstract

Steroid alkaloids have been suggested as potential anticancer compounds. However, the underlying mechanisms of how steroid alkaloids inhibit the tumor growth are largely unknown. Here, we reported that solanine, a substance of steroid alkaloids, has a positive effect on the inhibition of pancreatic cancer cell growth in vitro and in vivo. In pancreatic cancer cells and nu/nu nude mice model, we found that solanine inhibited cancer cells growth through caspase-3 dependent mitochondrial apoptosis. Mechanically, solanine promotes the opening of mitochondrial membrane permeability transition pore (MPTP) by downregulating the Bcl-2/Bax ratio; thereafter, Cytochrome c and Smac are released from mitochondria into cytosol to process the caspase-3 zymogen into an activated form. Moreover, we found that the expression of tumor metastasis related proteins, MMP-2 and MMP-9, was also decreased in the cells treated with solanine. Therefore, our results suggested that solanine was an effective compound for the treatment of pancreatic cancer.

## 1. Introduction

Pancreatic cancer is a malignant neoplasm, which causes the death of more than 30 thousand people per year in the United States [1]. Pancreatic cancer has extremely poor prognosis and less effective response to conventional therapy compared with other kinds of cancers. The 5-year relative survival rate of pancreatic cancer patients is about 6%, and the median survival time after diagnosis is up to 6 months [2–4]. Though the surgical resection of pancreatic cancer in the head is the most effective treatment, only 20% of the cases are surgically resectable [5]. Alternatively, a high proportion of pancreatic cancer patients have to be treated with radiation or chemotherapy rather than surgery. However, the survival rates in those nonsurgical pancreatic cancer patients were not changed. Thus, a novel effective therapeutic method or agent is needed for the treatment of those nonsurgical patients.

Solanine, one of the steroid alkaloids, belongs to the Solanaceae family. Solanine is mainly found in the tuber of potato (*Solanum tuberosum* L.) and the plant of nightshade (*Solanum nigrum *Linn.). The total alkaloids had a strong inhibitory effect on tumor growth in animals due to its cytotoxic effect on tumor cells [6, 7]. Further experiments showed that the ethanol extract of total alkaloids from ripe fruits exhibited proapoptotic effect on breast cancer cells [8]. This anticancer effect was further confirmed in another kind of steroid alkaloids in the family of Solanaceae [9].

As of now, the study of other compounds of Solanaceae family such as solanine is limited. To uncover the potential contribution of solanine to the cancer therapy and the potential mechanisms that underlie the relationships between solanine and tumorigenesis, the effect of solanine on pancreatic cancer was studied in this study.

## 2. Materials and Methods

### 2.1. Cell Lines and Nude Mice

Human pancreatic cancer cell lines, SW1990 and Panc-1 (Shanghai Institutes for Biological Sciences), were cultured in RPMI 1640 containing 10% FBS. Cell density was adjusted to 2 × 10^4^ cells per square centimeter before the addition of solanine (Sigma). Athymic nude nu/nu mice were obtained from the Shanghai Laboratory Animal Center at the Chinese Academy of Sciences, Shanghai, China. Animals were maintained at the Laboratory Animal Center of Wenzhou Medical University. The animal experiment protocol was approved by the Institutional Animal Committee of Wenzhou Medical University.

### 2.2. Cell Proliferation and Apoptosis Analysis

Cell proliferation was measured using the Cell Counting Assay Kit-8 (Dojindo Molecular Technologies, Kumamoto, Japan) according to the manufacturer's protocol. Briefly, 0.6 × 10^4^ cells were seeded in 96-well plate one day before the serum starvation procedure. After starving with the serum-free medium containing 0.1% BSA for 24 h, cells were treated with different concentrations of solanine for 24 h, 48 h, and 72 h as indicated. Then, cells were coincubated with Counting Assay Kit-8 solution for another 2 h. Cell viability was determined by measuring the absorbance at 450 nm using a plate reader (BioTek Instruments, USA). In the cells treated with solanine, the reduced percentages of cell viability compared with control cells without solanine were considered cell growth inhibition rates.

For the cell apoptosis analysis, cells with or without the treatment of solanine were stained with Annexin V/propidium iodide (PI) using Vybrant Apoptosis Assay Kit Number 2 (Molecular Probes). The apoptotic cells were analyzed by flow cytometry.

### 2.3. Quantitative Real-Time PCR

Total RNA were extracted from cultured cells or mice tissues with TRIzol Reagent (Ambion, NY, USA) following the manufacturer's instruction. The RNA quality was verified using spectrophotometric and agarose gel electrophoresis. Later on, the cDNA was synthesized with the ReverTra Ace qPCR RT kit (Toyobo, Tokyo, Japan) using 1 *μ*g RNA. Quantitative analyses of MMP-2, MMP-9, Bax, Bcl, p53, Smac, and Cytc mRNA expression were performed using SYBR Green Real-Time PCR Master Mix (Toyobo) on 7500 Real-Time PCR System (Applied Biosystems, Carlsbad, California, USA). The primers used here were listed in Table 1.

### 2.4. Immunoblotting

For the immunoblotting of P53, Bcl-2, Bax, and caspase-3, proteins were extracted from whole cell lysates using RIPA buffer (Cell Signaling) following manufacturer's instructions. For the immunoblotting of Cytochrome c and Smac, cytoplasmic proteins were extracted using NE-PER Nuclear Protein Extraction Kit (Thermo Fisher). The proteins separated by SDS gel were transferred to 0.22 *μ*m PVDF membrane (Bio-Rad) at 15 V for 2 h by using semidry transfer set (CBS Scientific). After blocking the membranes with 5% nonfat dry milk in Tris-buffered saline with Tween (TBST) (150 mM NaCl, 15 mM Tris-HCl (pH 7.5), and 0.1% Tween 20) for about 2 h, proteins were probed with primary antibody against Bcl-2 (Bioworld), caspase-3 (Bioworld), P53 (Bioworld), Bax (Bioworld), Smac/Diablo (Cell Signaling), Cytochrome c (Cell Signaling), or *β*-actin antibody (Santa Cruz) and then incubated with a secondary antibody conjugated with horseradish peroxidase (Cell Signaling). Membranes were washed by TBST after each antibody probing. Signals were detected by using Super Signal West Pico chemiluminescent substrate (Thermo Scientific). Integrated optical density (IOD) quantification was performed using Gel-Pro Analyzer 4.0.

### 2.5. ELISA

MMP-2 and MMP-9 levels in the supernatant of cell culture were determined using the ELISA kit according to the manufacturer's instructions.

### 2.6. In Vivo Tumorigenesis Assay

Around 4 × 10^6^ viable SW1990 cells were injected subcutaneously into 6-week-old nu/nu male nude mice. After 15 days of injection, the mice were divided into two groups (5/group) randomly and were fed with or without 5 mg/kg solanine as indicated for two weeks [10]. Tumors' sizes were measured daily in two dimensions with calipers. Furthermore, the mRNA levels of Bcl-2 and Bax were also determined by Real-Time PCR as described above.

### 2.7. Statistical Analysis

The data are presented as mean ± SD from three independent experiments. Statistical significance was evaluated by one-way ANOVA among groups of cells and independent Student's *t* test between groups in vivo with SPSS 13.0 software (IBM). *P* < 0.05 was considered statistically significant.

## 3. Results

### 3.1. Effect of Solanine in Cell Proliferation and Apoptosis

We found that solanine changed cell morphology in both SW1990 and Panc-1 cells. Cells without solanine had smooth cell membrane and elongated cell shape, while cells coincubated with solanine exhibited round and shrinking morphology in a dose-dependent manner.

**Figure 1:**
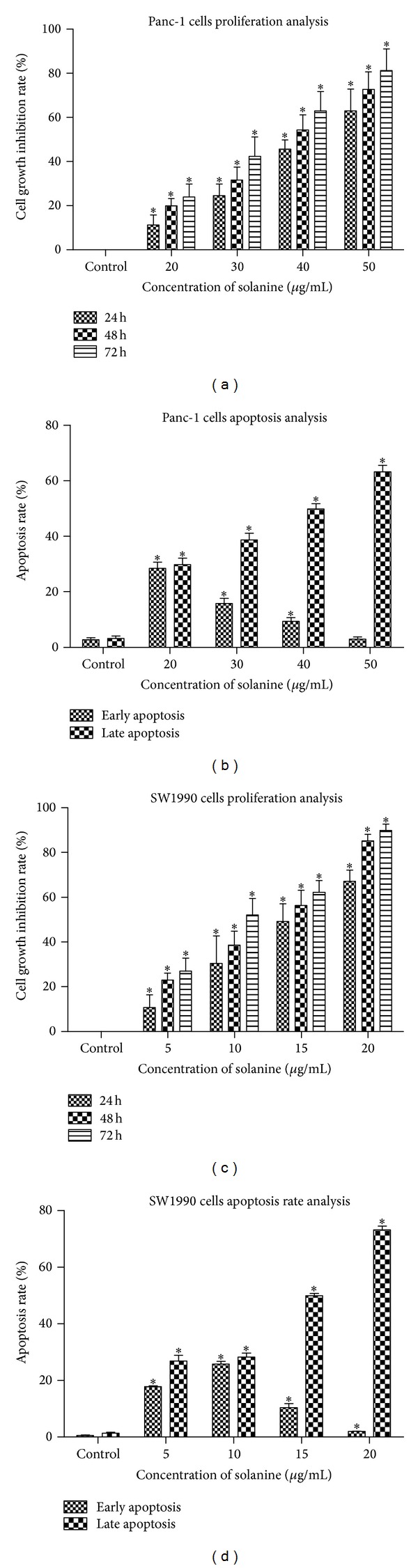
Solanine regulates pancreatic cancer cells viability. ((a) and (c)) After treatment with different solanine concentrations for 24 h, 48 h, and 72 h, cell growth inhibition rates were further calculated by determining the cell viability using CCK-8 in Panc-1 (a) and SW1990 (c) cells. ((b) and (d)) Apoptotic cells of Panc-1 (b) and SW1990 (d) cells were determined by treating the cells with different solanine concentration for 24 h. The early and late apoptosis were measured using Annexin V and PI based flow cytometry, respectively. Error bars, ±SD. *P < 0.05.

Then, we asked whether solanine can inhibit cell proliferation and promote cell apoptosis. In CCK8 based cell proliferation experiment, we found that solanine had a significant inhibitory effect on the growth of SW1990 and Panc-1 cell lines in a time- and dose-dependent manner (Figures 1(a) and 1(c)). In cell apoptosis study, we used Annexin V/propidium iodide (PI) based flow cytometry to test the early and late apoptosis in cells with and without solanine. We found that the numbers of total apoptotic cells were increased when the SW1990 and Panc-1 cells were treated with solanine for 24 h (Figures 1(b) and 1(d)). Furthermore, an increased ratio of late apoptotic cells to early apoptotic cells was observed when the dosage of solanine in the culture of SW1990 and Panc-1 cells increased (Figures 1(b) and 1(d)). It suggested that the apoptotic effect was dose-dependent.

### 3.2. Solanine Regulate Mitochondria-Mediated Cell Apoptosis and Tumor Metastasis

**Figure 2:**
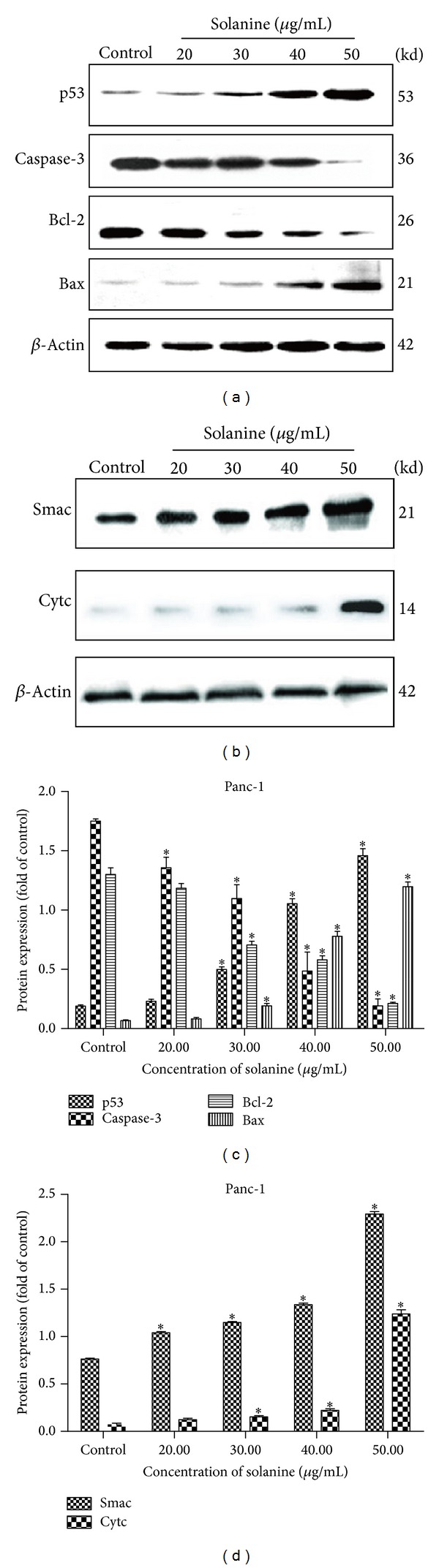
Solanine regulates mitochondrion mediated Panc-1 cell apoptosis. Immunoblot analysis of apoptosis related protein, Bcl-2, Bax, P53, caspase-3, Cytc, and Smac protein in Panc-1 cells. ((a) and (b)) Immunoblots analysis of whole cell lysates from Panc-1. Antibodies were used as indicated. ((c) and (d)) Immunoblots analysis of cytoplasmic protein extraction from Panc-1. Antibodies were used as indicated. Error bars, ±SD. *P < 0.05.

**Figure 3:**
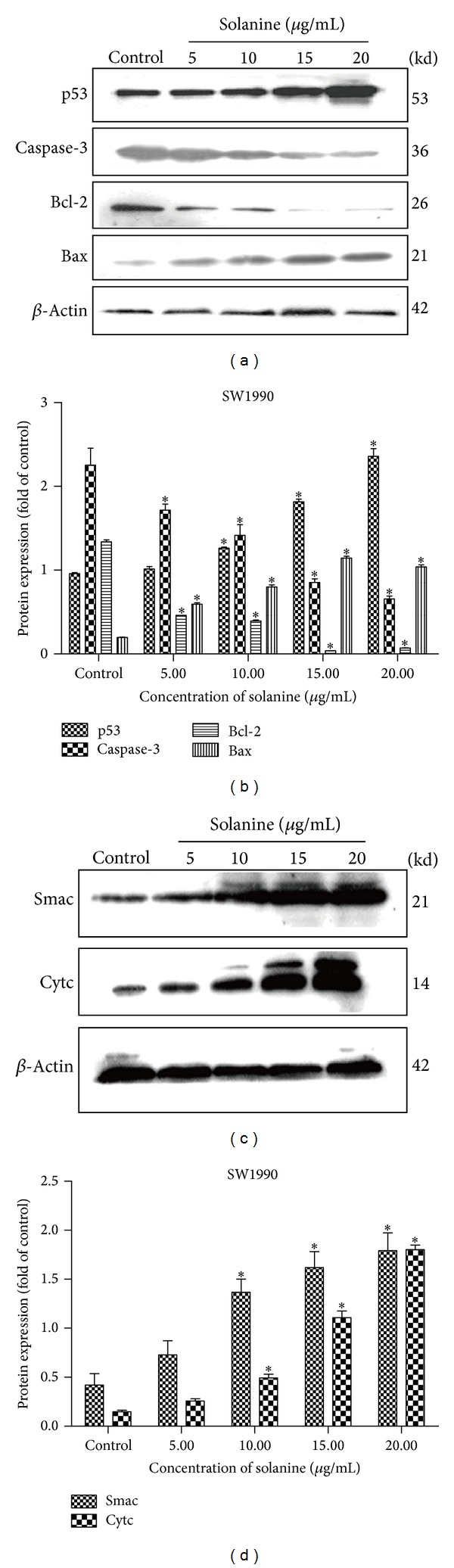
Solanine regulates mitochondrion mediated SW1990 cell apoptosis. Immunoblot analysis of apoptosis related protein, Bcl-2, Bax, P53, caspase-3, Cytc, and Smac, in SW1990 cells. ((a) and (b)) Immunoblots analysis of whole cell lysates from SW1990. Antibodies were used as indicated. ((c) and (d)) Immunoblots analysis of cytoplasmic protein extraction from SW1990. Antibodies were used as indicated. Error bars, ±SD. *P < 0.05.

**Figure 4:**
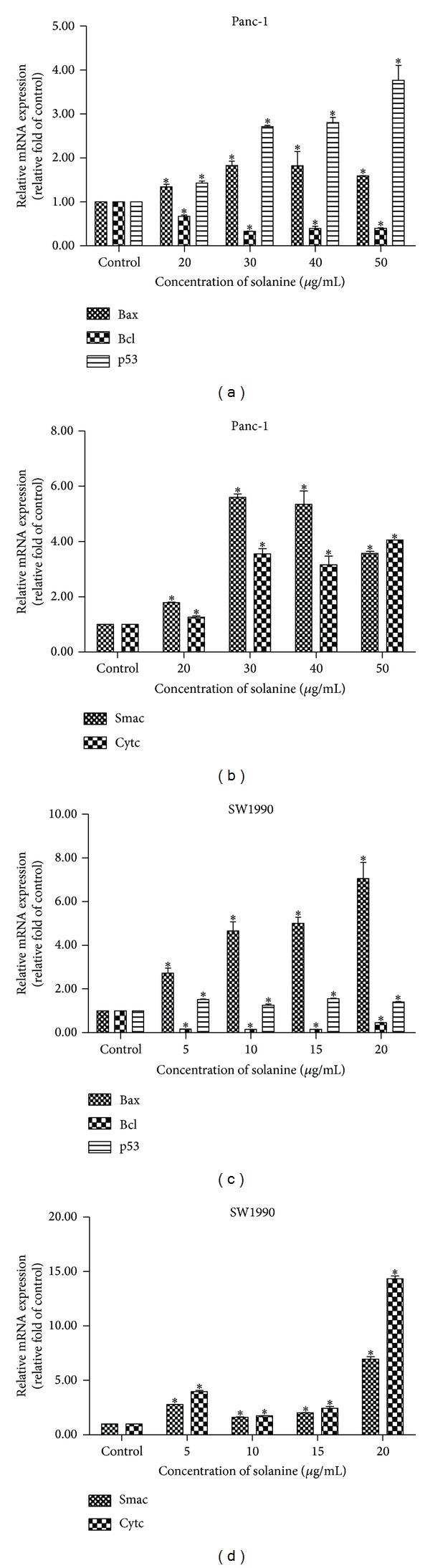
Solanine regulates mitochondrion mediated pancreatic cancer cells apoptosis. Quantitative RT-PCR analysis of apoptosis related mRNA level, Bcl-2, Bax, P53, Cytc, and Smac, in Panc-1 ((a) and (b)) and SW1990 ((c) and (d)) cells.

To investigate the possible mechanism of solanine in the apoptosis of pancreatic cancer cells, we measured the abundance of several apoptosis related proteins in cells with and without solanine treatment (Figures 2 and 3). In Panc-1 (Figures 2(a) and 2(b)) and SW1990 (Figures 3(a) and 3(b)) cells, we found the cells treated with solanine containing higher P53 and Bax and lower Bcl-2 levels, which means that the mitochondrial membrane permeability transition pore (MPTP) was opened in these cells due to the decreased Bcl-2/Bax ratio. As a consequence, the increased Cytochrome c and Smac levels were found in cytosol (Figures 2(c), 2(d), 3(c), and 3(d)). These results were further confirmed at mRNA level in Panc-1 (Figures 4(a) and 4(b)) and SW1990 cells (Figures 4(c) and 4(d)) with and without solanine. To confirm that the release of Cytochromes c and Smac from mitochondria into cytosol plays an initial role in mitochondrial apoptosis, we verified the expression of downstream proteins of Cytochrome c. As shown in Figures 2(a), 2(b), 3(a), and 3(b), we found that the caspase-3 zymogen level was decreased in whole cells. Taken together, our results demonstrated that a caspase-3 dependent mitochondria apoptosis was activated in pancreatic cells treated with solanine.

**Figure 5:**
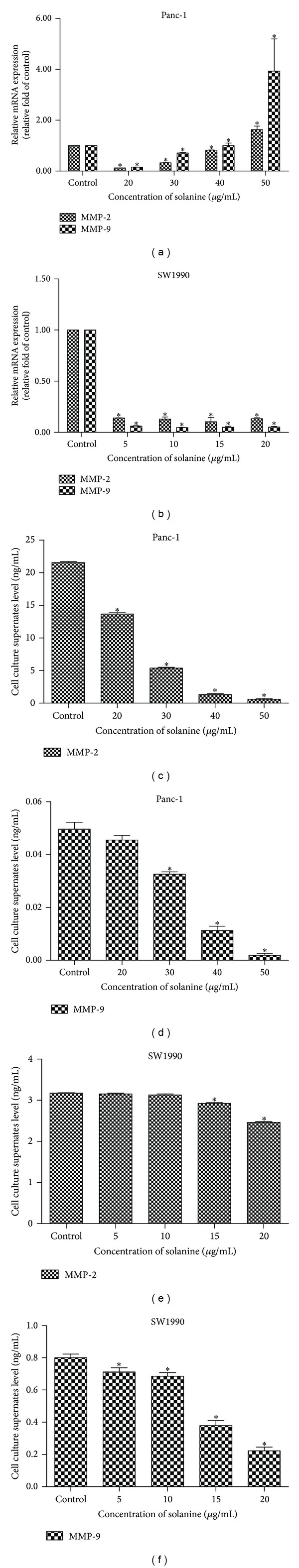
Solanine inhibits tumor metastasis ((a) and (b)). After 24 h of treatment of different solanine concentration, the mRNA level of MMP-2 and MMP-9 in Panc-1 (a) and SW1990 (b) was determined by quantitative RT-PCR. ((c)–(f)) After 24 h of treatment of different solanine concentrations, the protein level of MMP-2 and MMP-9 in Panc-1 ((c) and (d)) and SW1990 ((e) and (f)) cells was measured by ELISA. Error bars, ±SD. *P < 0.05.

To further test whether solanine can affect tumor metastasis, we determined the level of tumor metastasis related marker proteins, MMP-2 and MMP-9, in Panc-1 and SW1990 cells. Quantitative RT-PCR showed that the mRNA levels of MMP-2 and MMP-9 were decreased in Panc-1 (Figure 5(a)) and SW1990 cells (Figure 5(b)) treated with solanine. At protein level, we found that the expression levels of MMP-2 and MMP-9 were upregulated in a dose-dependent manner in the supernatant of Panc-1 (Figures 5(c) and 5(d)) and SW1990 (Figures 5(e) and 5(f)) cells treated with solanine. These results suggested that solanine can regulate the tumor metastasis in pancreatic cancer.

### 3.3. Solanine Inhibits Tumorigenicity

**Figure 6:**
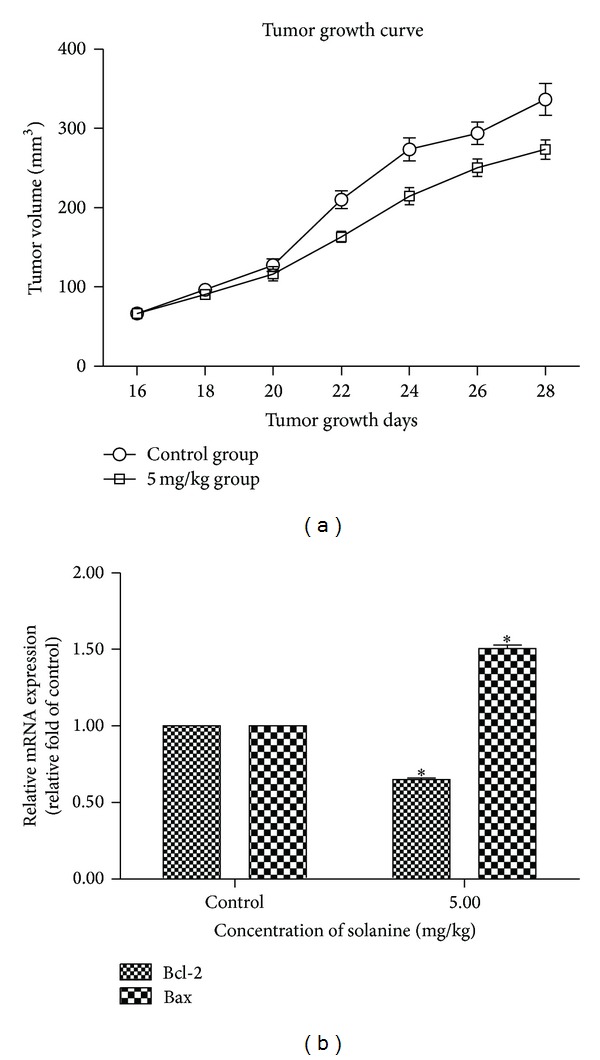
Solanine suppressed tumor growth in a xenograft model of pancreatic cancer. (a) 4 × 10^6^ SW1990 cells were injected into nude mice. Three independent experiments with five mice in each group were used in this experiment. After 15 days of injection, mice were fed with solanine at 5 mg/kg. After each 2 days, tumor size was measured in the mice fed with or without solanine. (b) Quantitative RT-PCR results of Bcl-2 and Bax in the xenograft tumor derived cells. Error bars, ±SD. *P < 0.05.

To investigate the influence of solanine in the tumor formation of pancreatic cancer, we injected 4 × 10^6^ SW1990 cells into nu/nu nude mice. The mice treated with 5 mg/kg solanine resulted in a dramatic reduction of tumor size (Figure 6(a)). In the mice fed with solanine, an increased Bax and a decreased Bcl-2 level were found at both mRNA (Figure 6(b)) and protein levels (Table 2). Thus, our results indicated that solanine has an inhibitory effect on tumor growth in vivo, which is probably through mitochondria-mediated cell apoptosis.

## 4. Discussion

Previous studies showed that steroid alkaloids, such as solasonine, solamargine, and solanine, exhibited an anticancer effect due to their roles in the apoptosis of cancer cells [11–16]. To date, no such reports were found in pancreatic cancer. In this study, we first observed the cytotoxic effect of solanine in two human pancreatic cancer cell lines, Panc-1 and SW1990, by using cell proliferation test and apoptosis assay. Further investigation showed that solanine bears an anticancer effect via the regulation of mitochondria-mediated cell apoptosis.

As we all know, apoptosis occurs in two-principal pathways: the mitochondria-mediated pathway and the death receptor-mediated pathway [17, 18]. The death receptor-mediated pathway is triggered by the binding of death-inducing ligands to cell surface receptors. And the mitochondria-mediated pathway is triggered by a variety of apoptotic stimuli in mitochondria, which increases the permeability of mitochondrial membrane and causes the release of Cytochrome c and Smac into the cytoplasm [19–22]. It has been known that the ratio of Bcl-2 to Bax determines the response to a death signal via modulating the mitochondrial membrane permeability transition (MTP) pore formation [23]. Decreased Bcl-2 loses the ability to inhibit the MTP pore formed by Bax complex, while increased Bax promotes the pore formation directly [23]. In our study, we found a decreased Bcl-2 and an increased Bax in solanine treated pancreatic cells. It indicated that solanine might activate mitochondria-mediated apoptosis by increasing the permeability of the mitochondrial membrane. Moreover, we found solanine treated cells containing higher Cytochrome c and Smac level in cytoplasmic and less of full length inactivated caspase-3 in whole cells [19]. Thus, we speculated that solanine can induce caspase-3 dependent mitochondrial apoptosis, in which solanine increases the permeability of mitochondrial membrane and releases Cytochrome c and Smac from mitochondria into cytosol to activate the caspase-9. Caspase-9 can then go on to activate caspase-3 by truncating the full length inactivated caspase-3 into activated forms, which are responsible for the cell apoptosis. Furthermore, our suggestion was further validated by checking the level of P53, which was found to be upregulated in caspase-3 dependent mitochondrial apoptosis [24]. Besides, our results indicated that solanine can also inhibit the process of metastasis by downregulating the expression of cancer cells migration related proteases, MMP-2 and MMP-9 [25, 26].

In conclusion, we demonstrated the mechanism of solanine as a cancer inhibitor in pancreatic cancer cell lines, by which we hope to give a new sight into the importance of solanine in the treatment of pancreatic cancer. However, further investigations are still needed to carefully clarify the side effects.

## Figures and Tables

**Table 1:** Primer pairs used in RT-PCR in vitro and in vivo.

Genes	Primers (5′-3′)	BP
In vitro		
Bax	Forward ACAAAGATGGTCACGGTCTGCC	242
	Reverse ACCAAGAAGCTGAGCGAGTGTC	
Bcl-2	Forward GCTCT TCAGG GACGG GGT	166
	Reverse GACAG CCAGG AGAAA TCAAA CAG	
P53	Forward TTCCG AGAGC TGAAT GAGGC	435
	Reverse TTTTT ATGGC GGGAG GTAGA CT	
Cytc	Forward AGACA TGGAG ACCAA AATCA AGAAC	132
	Reverse CTCCT TTAGC GGTCA TTGCC	
Smac	Forward AGCTG GAAAC CACTT GGATGA	138
	Reverse GAATG TGATT CCTGG CGGTTA	
MMP-2	Forward AATGC CATCC CCGAT AACC	397
	Reverse GCTCA GCAGC CTAGC CAGTC	
MMP-9	Forward GGGGG AAGAT GCTGC TGTT	440
	Reverse AGCGG TCCTG GCAGA AATAG	
GAPDH	Forward GTCTT CACCA CCATG GAGAA	267
	Reverse ATCCA CAGTC TTCTG GGTGG	

In vivo		
Bax	Forward TGGCAGCTGACATGTTTTCTGAC	195
	Reverse CGTCCCAACCACCCTGGTCT	
Bcl-2	Forward GTCATGTGTGTGGAGAGCGT	144
	Reverse GCCGTACAGTTCCACAAAGG	
Caspase-3	Forward CAGACAGTGGAACTGACGAT	152
	Reverse TTTCAGCATGGCGCAAAGTG	
GAPDH	Forward GGTGGAAGGTCGGTGTGAACG	234
	Reverse CTCGCTCCTGGAAGATGGTG	

**Table 2:** The immunohistochemistry analysis of Bax, caspase-3, and Bcl-2 in tumor xenografts.

Groups	*n *	Bax		Bcl-2		*P* value
		+	−	+	−	
Control group	50	19	11	39	31	
5 mg/kg group	50	29	20	30	21	<0.05

*P* value is of the control group versus solanine groups.
